# Identification and functional characterization of a flavonol synthase gene from sweet potato [*Ipomoea batatas* (L.) Lam.]

**DOI:** 10.3389/fpls.2023.1181173

**Published:** 2023-05-10

**Authors:** Meng Kou, Chen Li, Weihan Song, Yifan Shen, Wei Tang, Yungang Zhang, Xin Wang, Hui Yan, Runfei Gao, Muhammad Qadir Ahmad, Qiang Li

**Affiliations:** ^1^ Xuzhou Institute of Agricultural Sciences in Jiangsu Xuhuai District/Sweet Potato Research Institute, Chinese Academy of Agricultural Sciences/Key Laboratory of Biology and Genetic Breeding of Sweet Potato, Ministry of Agriculture and Rural Affairs, Xuzhou, China; ^2^ Department of Plant Breeding and Genetics, Bahauddin Zakariya University, Multan, Pakistan

**Keywords:** sweet potato, flavonol synthase, flavonols, anthocyanins, RNAi

## Abstract

Flavonol synthase (FLS) is a key enzyme of the flavonoid biosynthetic pathway, which catalyzes the conversion of dihydroflavonols into flavonols. In this study, the FLS gene *IbFLS1* was cloned and characterized from sweet potato. The resulting IbFLS1 protein showed a high similarity with other plant FLSs. The conserved amino acids (HxDxnH motifs) binding ferrous iron and residues (RxS motifs) binding 2-oxoglutarate were found in IbFLS1 at conserved positions, as in other FLSs, suggesting that IbFLS1 belongs to the 2-oxoglutarate-dependent dioxygenases (2-ODD) superfamily. qRT-PCR analysis showed an organ-specific pattern of expression of the *IbFLS1* gene, which was predominantly expressed in young leaves. The recombinant IbFLS1 protein could catalyze the conversion of dihydrokaempferol and dihydroquercetin to kaempferol and quercetin, respectively. The results of subcellular localization studies indicated that IbFLS1 was found mainly in the nucleus and cytomembrane. Furthermore, silencing the *IbFLS* gene in sweet potato changed the color of the leaves to purple, substantially inhibiting the expression of *IbFLS1* and upregulating the expression of genes involved in the downstream pathway of anthocyanin biosynthesis (i.e., *DFR*, *ANS*, and *UFGT*). The total anthocyanin content in the leaves of the transgenic plants was dramatically increased, whereas the total flavonol content was significantly reduced. Thus, we conclude that *IbFLS1* is involved in the flavonol biosynthetic pathway and is a potential candidate gene of color modification in sweet potato.

## Introduction

Flavonoids belong to polyphenolic secondary metabolites, are widely present in plant tissues, and can be classified into various subclasses (flavanols, dihydroflavonols, flavonoids, flavonols, flavanols, anthocyanins, proanthocyanidins, and isoflavones) depending on the modification of the C-ring (Iwashina, 2000). Flavonols are the largest subgroup of flavonoids, consisting mainly of kaempferols, quercetins, and myricetins (Jiang et al., 2020). They play pivotal roles in plant growth and development, such as regulating auxin transport (Kuhn et al., 2011), against UV light (Majer et al., 2014), alleviating oxidative stress (Redha et al., 2012), and influencing color formation (Tian et al., 2015). Flavonols have also been demonstrated to have pharmacological functions including anti-inflammatory, anti-angiogenesis, anti-oxidation, antiproliferative, and cardioprotection functions (Harborne and Williams, 2000; Lee et al., 2005; Kim et al., 2006). Flavonoids are synthesized *via* the phenylpropane metabolic pathway (Czemmel et al., 2009). Flavonol synthase (FLS) is an important enzyme in the flavonoid metabolism; it catalyzes the conversion of dihydroflavonols (dihydroquercetin, dihydrokaempferol, and dihydromyricetin) into relative flavonols (quercetin, kaempferol, and myricetin) (Czemmel et al., 2009). At this stage, dihydroflavonol 4-reductase (DFR) competes with FLS for the common substrates dihydroflavonols and produces anthocyanins. Therefore, the *FLS* gene has great importance in the downstream branch of the flavonoid pathway, affecting not only flavonol synthesis, but also anthocyanin accumulation and plant coloration (Luo et al., 2016).

The first *FLS* gene was isolated from *Petunia hybrida* and functionally verified in yeast and plants (Holton et al., 1993). To date, a number of *FLS* genes have been cloned and characterized in other plant species, such as *Arabidopsis thaliana* (Pelletier et al., 1997), *Citrus unshiu* (Moriguchi et al., 2002), *Zea mays* (Ferreyra et al., 2010), *Fagopyrum tataricum* (Li et al., 2012), *Litchi chinensis* (Liu et al., 2018), and *Camellia sinensis* (Shi et al., 2021).

The sweet potato [*Ipomoea batatas* (L.) Lam.] is one of the most important food crops due to its wide adaptability, high nutritive value, high yield potential, and low input requirements (Park et al., 2020; Yan et al., 2022). It is ranked sixth worldwide in terms of total crop production. Purple-fleshed sweet potato (PFSP) is a special kind of sweet potato. In addition to containing a variety of micronutrients and minerals, PFSP is also rich in natural anthocyanins with health-promoting effects (Esatbeyoglu et al., 2017; Jang et al., 2019); therefore, it is welcomed by more and more consumers. To date, research on PFSP anthocyanin has mainly focused on anthocyanin metabolism and the regulation mechanism, while research on the effect of competition for the flavonol branch on anthocyanin synthesis and color formation is lacking. In the present study, cDNA of sweet potato *FLS* (*IbFLS1*) was isolated from the commercial dark-purple-fleshed sweet potato cultivar ‘Xuzi 8’ based on our previous transcriptome data. The identification and characteristic of *IbFLS1* can provide valuable information about the flavonoid biosynthetic pathway and lay the foundation for improving nutritional value in sweet potato plants.

## Materials and methods

### Plant materials

The dark-purple-fleshed sweet potato cultivar ‘Xuzi 8’ was planted in the field of Xuzhou Institute of Agricultural Sciences in Jiangsu Xuhuai District. Young leaf (YL), leaf petiole (LP), stem (S), fibrous root (FR), pencil root (PR), and storage root (SR) were collected 120 days after transplanting. All tissues were frozen immediately in liquid nitrogen and stored at –80°C until total RNA extraction and flavonol determination

### Total RNA extraction and the first-strand cDNA synthesis

Total RNAs of all samples were extracted using the polysaccharide polyphenol plant RNA Isolation Kit (Huayueyang Biotechnology Co., Ltd., Beijing, China) following the manufacturer’s protocol. RNA integrity and concentration were verified by, respectively, 1.2% formaldehyde denaturing agarose gel electrophoresis and a NanoDrop 1000 spectrophotometer (ThermoFisher Scientific Inc., Waltham, MA, USA). The cDNA was synthesized using ReverTra Ace^®^ qPCR RT Master Mix with a gDNA Remover kit (Toyobo, Osaka, Japan) in accordance with the manufacturer’s instructions.

### Cloning of *IbFLS1*

An *FLS* homolog was identified from the previous transcriptome data and designated *IbFLS1*. The coding sequence (CDS) of *IbFLS1* was amplified by PCR from ‘Xuzi 8’ SR cDNA. Gene-specific primers (IbFLS1-1-F and IbFLS1-1-R; see **Supplementary Table S1**) and PrimeSTAR^®^ Max DNA Polymerase (Takara Biomedical Technology Co., Ltd., Beijing, China) were used for this experiment under the following conditions: 30 cycles of 98°C for 10 s, 55°C for 15 s, and 72°C for 30 s and a final extension at 72°C for 10 min. The PCR-amplified product was cloned into a pEASY-Blunt vector (TransGen Biotech Co., Ltd, Beijing, China), transformed into *Escherichia coli* (*E. coli*) Trans1-T1 (TransGen Biotech Co., Ltd, Beijing, China), and then sequenced at Sangon Biotech (Shanghai) Co., Ltd. (China).

### Sequence alignment and phylogenetic analysis of IbFLS1

The amino acid sequences of IbFLS1 and other FLS proteins were obtained from the GenBank database. Multiple sequence alignments were performed with DNAMAN software. A phylogenetic tree was constructed by the maximum likelihood method with 1,000 bootstrap replicates using MEGA 6.0.

### Gene expression analysis by quantitative real-time PCR (qRT-PCR)

cDNAs of all samples were diluted 10-fold for qRT-PCR analysis. qRT-PCR primers (IbFLS1-2-F and IbFLS1-2-R) of the *IbFLS1* gene were designed according to the full-length sequence (primers are listed in **Supplementary Table S1**), and *IbARF* was used as the internal control gene (Park et al., 2012). The mRNA levels were quantified by qRT-PCR amplification using a StepOnePlus™ real-time PCR system (ABI, USA) in a total volume of 20 μL, containing 10 μL of SYBR^®^ Green Realtime PCR Master Mix (TOYOBO, Osaka, Japan), 2 μL of cDNA templates, 1 μL of forward and reverse primer (10 mM), and 7 μL of ddH_2_O. The reaction program was performed as presented in a previous study (Kou et al., 2019). Gene transcript levels were calculated using the 2^–ΔΔCT^ method (Livak and Schmittgen, 2001), and each reaction was performed in triplicate.

### Expression of *IbFLS1* in *E. coli* and enzyme activity assay

Using the pEASY-Blunt : IbFLS1 vector as a template, the forward primer (IbFLS1-3-F) and reverse primer (IbFLS1-3-R) were separately designed with *Nde*I and *Bam*HI restriction sites (primers are listed in **Supplementary Table S1**) to construct the pET-28a(+):IbFLS1 vector, as mentioned in the previous study (Kou et al., 2019). The recombinant protein expression and purification method used were reported previously by Kou et al. (2019). The enzyme activity of IbFLS1 was measured in international enzyme units (IU), as described by Li et al. (2012). One IU is the amount of FLS that produces 1 μmol of quercetin or kaempferol per minute (Prescott and John, 1996).

### Subcellular localization of IbFLS1

Using the pEASY-Blunt : IbFLS1 vector as a template, the coding region of IbFLS1 without a termination codon was inserted between the *Sal*I and *Spe*I sites of the pCAMBIA1301S vector (replacing GUS with GFP),using T4 DNA ligase to generate a pCAMBIA1301S-GFP : IbFLS1 fusion construct (primers are listed in **Supplementary Table S1**). The transient GFP fusion vector was infiltrated into tobacco leaves *via* an *Agrobacterium*-mediated infiltration method (You et al., 2022), and the transformed leaves were monitored with a laser confocal microscope (Nikon C2).

### Expression vector construction and stable sweet potato transformation

Using the pEASY-Blunt : IbFLS1 vector as a template, the FS and RS parts of IbFLS1-RNAi were cloned with FLS1i-F(*Kpn*I)/FLS1i-R(*Cla*I) and FLS1i-F(*Bam*HI)/FLS1i-R(*Xho*I) (see **Supplementary Table S1**). First, the FS part of IbFLS1-RNAi was inserted between the *Kpn*I and *Cla*I sites of the PBS-RNAi vector (modified intermediate vector), using the T4 DNA ligase to generate the recombinant vector PBS-RNAi-FS. Then, the RS part of IbFLS1-RNAi was inserted between the *BamH*I and *Xho*I sites of PBS-RNAi-FS to generate the fusion construct PBS-RNAi-FS/RS. Finally, the pCAMBIA1301S-IbFLS1-RNAi vector was successfully created by linking PBS-RNAi-FS/RS and pCAMBIA1301S (with *Kpn*I and *Bam*HI cutting sites) together. Subsequently, the IbFLS1:RNAi vector was introduced into ‘Xuzi 8’ *via Agrobacterium tumefaciens*-mediated transformation, as described previously (You et al., 2022).

### Determination of flavonol and anthocyanin

The fresh samples were ground to powder in liquid nitrogen, transferred to the centrifugal tube, and 10 mL of extracting solution (V_methanol_ : V_acetone_:V_amylalcohol_ = 2:2:0.5) was added. After adding the glass ball, the ultrasonic extraction was performed for 20 min (100 W), followed by heating for 5 min in a microwave oven (200 W), and then filtered through 0.22-µm Millipore filters. LC-MS analysis was carried out with a TripleTOF^®^ 4600 System (AB SCIEX, USA) connected to a HALO-C18 column (2.7 μm, 100 × 4.6 mm; AMT, USA). The mobile phase consisted of 0.5% (v/v) formic acid (A) and acetonitrile containing 0.5% (v/v) formic acid (B). The gradient profile was optimized as follows: 0 min, 95% A/5% B; 10 min, 30% A/70% B; 15 min, 0 A/100% B; 20 min, 0% A/100% B; 25 min, 95% A/5% B. The flow rate was 0.5 mL/min. Then the processed sample was analyzed by a tandem mass spectrometer with an electrospray ion source (EIS), monitored under multiple reaction monitoring (MRM) mode. The standard curve and regression equation were established (quercetin: *y* = 410,287*x* – 10,152.3367, *R* ^2^ = 0.9984; kaempferol: *y* =257,442*x* – 16,761.2444, *R* ^2^ = 0.9997; myricetin: y = 716,220*x* – 40,014.6274, *R* ^2^ = 0.9949). The total flavonol content was the sum of quercetin, kaempferol, and myricetin content. The total anthocyanin content of sweet potato tissues was measured in accordance the method proposed by Guo et al. (2015) and calculated using the equation Q_Anthocyanin_ = (A530–0.25 × A657) × 0.1 M^–1^. All samples were performed in three biological replicates.

### Statistical analysis

Significant differences between treatments and multiple comparisons were analyzed using Microsoft Office Excel 2010, and all data are represented as the mean ± standard deviation (SD).

## Results

### Cloning and sequence analysis of *IbFLS1*

**Figure 1:**
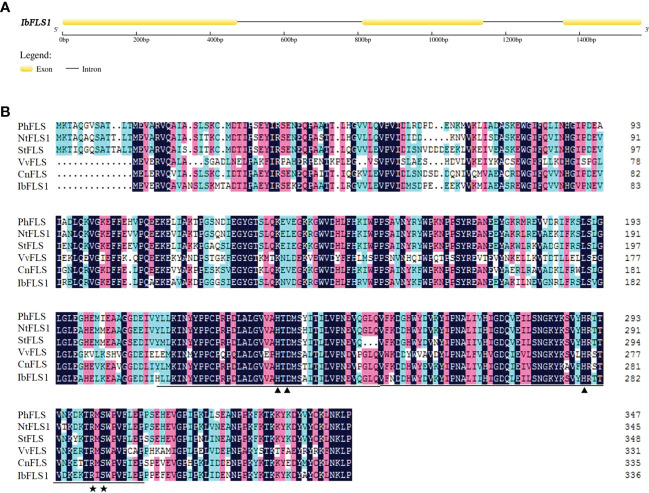
Analysis of the *IbFLS1* gene structure. **(A)**, intron analysis of the *IbFLS1* gene; **(B)**, alignment of IbFLS1 with other FLS proteins. The Fe^2+^-binding sites and oxoglutarate binding sites are indicated by black arrowheads and asterisks, respectively, and the conserved Fe^2+^/2-ODD domain is underlined. *Petunia hybrida* (PhFLS, CAA80264.1), *Nicotiana tabacum* (NtFLS1, ABE28017.1), *Solanum tuberosum* (StFLS, CAA63092.1), *Vitis vinifera* (VvFLS, BAE75810.1), *Camellia nitidissima* (CnFLS, ADZ28516.1).

In this study, we used already available transcriptome data and explored a *FLS* homolog designated *IbFLS1*. The CDS of *IbFLS1* was isolated from the sweet potato cultivar ‘Xuzi 8’, and was found to encode 337 amino acids with a molecular weight of 38.17 kDa and a theoretical pI of 5.70 analyzed online by the ProtParam tool (http://web.expasy.org/protparam/). The genomic DNA of *IbFLS1* was also isolated, and was found to consist of 1,568 nucleotides, including two introns and three exons (**Figure 1A**).

Comparison of the deduced amino acid sequence of IbFLS1 with other FLS proteins revealed that IbFLS1 possessed the typical conserved HxDxnH motifs (His223, Asp225, and His279) for binding ferrous iron and RxS motifs (Arg289 and Ser291) for binding 2-oxoglutarate (**Figure 1B**). This indicated that IbFLS1 belongs to the soluble Fe(II)- and 2-oxoglutarate-dependent dioxygenases (2-ODD) superfamily. Therefore, it is inferred that IbFLS1 protein plays a similar function to other plant FLS proteins in flavonol synthesis.

**Figure 2:**
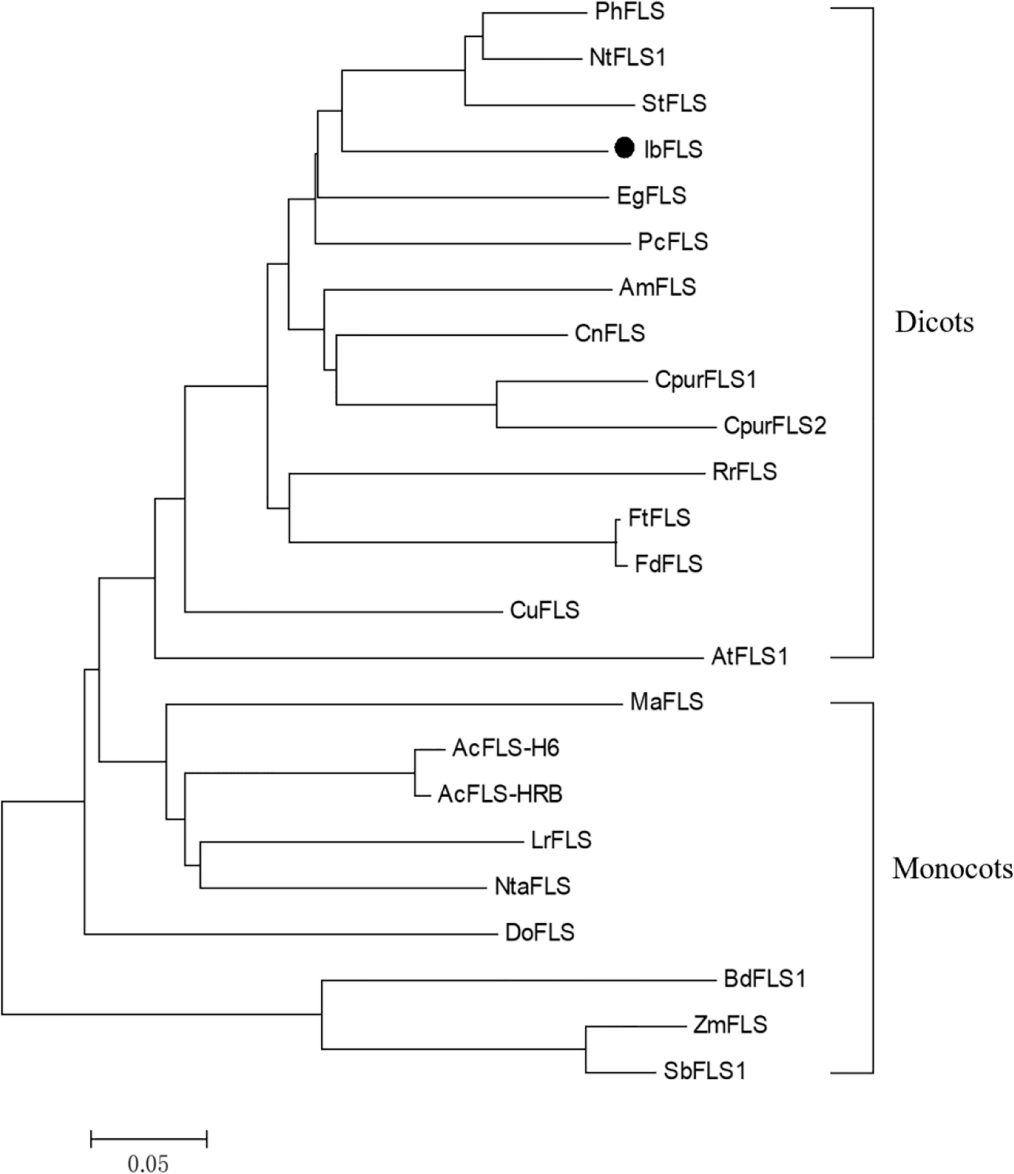
Phylogenetic tree of IbFLS1 and other FLS proteins. *Arabidopsis thaliana* (AtFLS1, AAB41504.1), *Petunia hybrida* (PhFLS, CAA80264.1), *Nicotiana tabacum* (NtFLS1, ABE28017.1), *Solanum tuberosum* (StFLS, CAA63092.1), *Camellia nitidissima* (CnFLS, ADZ28516.1), *Citrus unshiu* (CuFLS, BAA36554.1), *Cyclamen purpurascens* (CpurFLS1 and CpurFLS2, BBA27023.1 and BBA27024.1), *Rosa rugosa* (RrFLS, KM099095), *Fagopyrum tataricum* (FtFLS, AEC33116.1), *Fagopyrum dibotrys* (FdFLS, AHN19765.1), *Antirrhinum majus* (AmFLS, ABB53382.1), *Eustoma grandiflorum* (EgFLS, AAF64168), *Petroselinum crispum* (PcFLS, AAP57395.1), *Muscari aucheri* (MaFLS, QBO54037.1), *Dendrobium officinale* (DoFLS, ATD53725.1), *Allium cepa* (AcFLS-H6 and AcFLS-HRB, AAO63023.1 and AAT68476.1), *Zea mays* (ZmFLS, XP_008646309.1), *Lilium regale* (LrFLS, ASV46329.1), *Narcissus tazetta* (NtaFLS, AFS63900.1), *Brachypodium distachyon* (BdFLS1, XP_003570562.1), *Sorghum bicolor* (SbFLS1, XP_002454608.1). The scale bar represents genetic distance.

BLASTP alignment of the amino acid sequence of IbFLS1 in NCBI showed that the IbFLS1 protein had the highest homology with *Ipomoea triloba* (98.81%), followed by *Ipomoea nil* (95.25%). To further investigate the relationship between the IbFLS1 protein and other plant FLSs, we constructed a phylogenetic analysis using functionally characterized plant FLSs (**Figure 2**). The phylogenetic tree showed that plant FLSs could be classified into two distinct clades: dicotyledonous and monocotyledonous. IbFLS1 protein belonged to the dicotyledonous clade, which is closely related to PhFLS (*Petunia hybrida*), NtFLS1 (*Nicotiana tabacum*), and StFLS (*Solanum tuberosum*), and then clustered together with EgFLS (*Lilium regale*) and PcFLS (*Petroselinum crispum*). These results demonstrate that the phylogenetic analysis matched well with the genetic relationships among the plant species.

### Tissue-specific expression analysis of the *IbFLS1* gene

**Figure 3:**
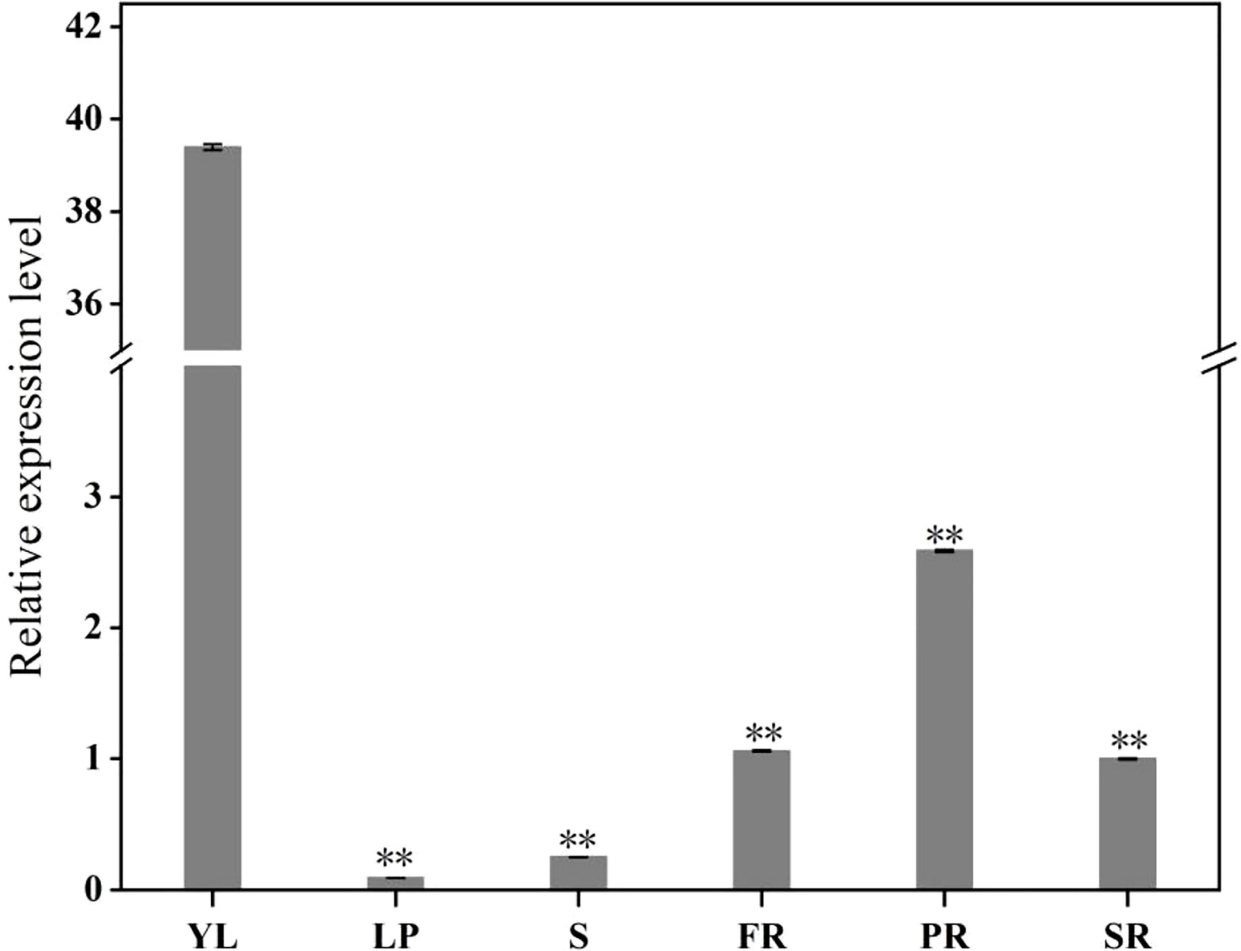
The expression profiles of *IbFLS1* in different tissues. Young leaf (YL), leaf petiole (LP), stem (S), fibrous root (FR), pencil root (PR), and storage root (SR). Each bar represents the mean ± SD) of three independent replicates. Significant differences between means are indicated by asterisks (**p < 0.01) above the bars. The significance analysis was carried out using YL as the control.

To study the expression levels of the *IbFLS1* gene in different tissues, qRT-PCR was used to detect the expression pattern (**Figure 3**). The results indicated that *IbFLS1* expression was significantly high in YL, and markedly lower in PR, SR, and FR, whereas *IbFLS1* expression was barely detectable in leaf petiole (LP) and stem (S).

### Recombinant IbFLS1 protein exhibits bifunctional activity

**Figure 4:**
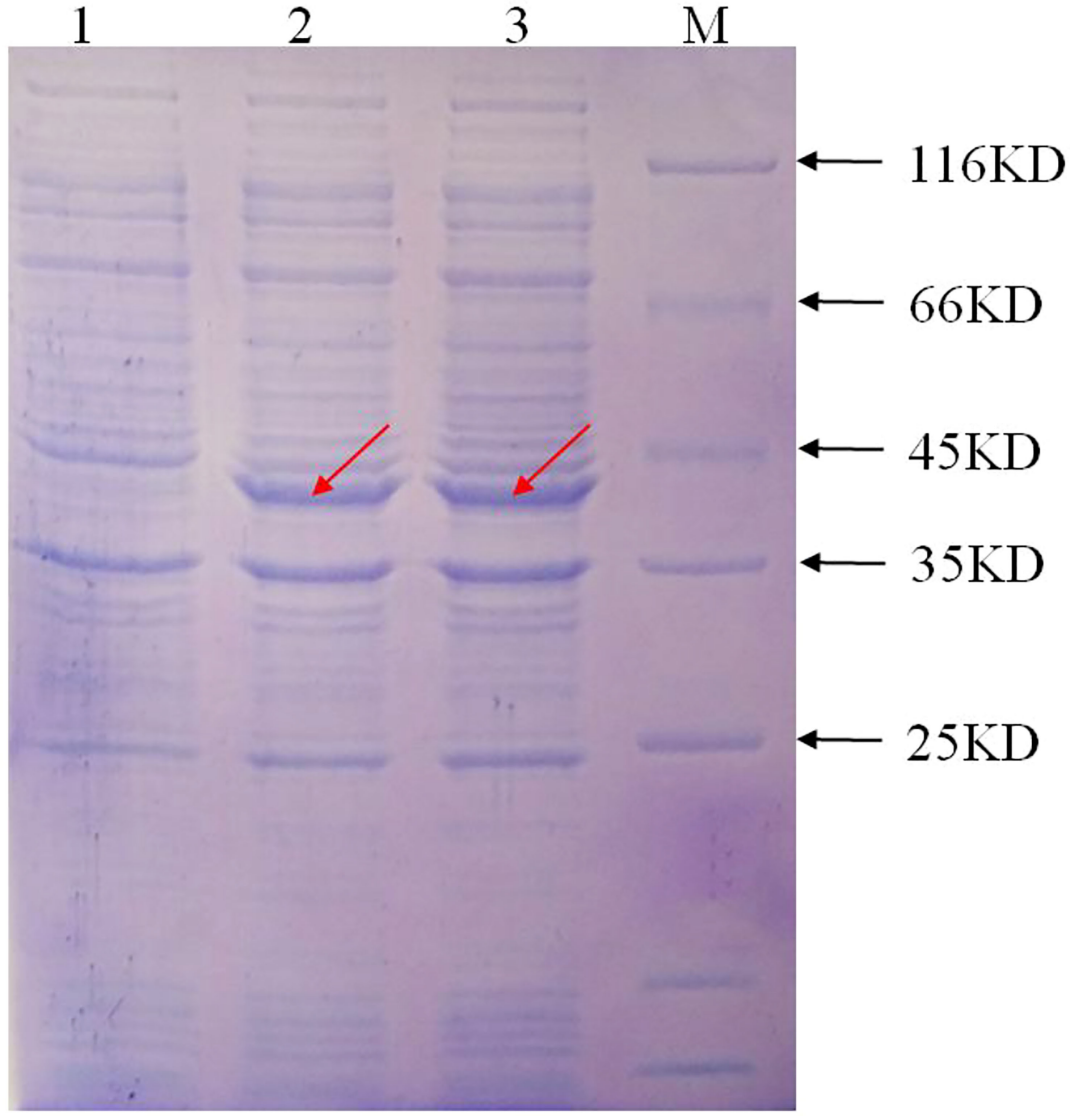
The expression of the recombinant protein induced by IPTG. M, protein marker; lane 1, the recombinant protein without IPTG induction; lane 2 and lane 3, the recombinant protein with IPTG induction, respectively. The red arrow represents the protein band induced by IPTG.

The prokaryotic expression vector pET-28a(+): IbFLS1 was transformed into *E. coli*, induced by isopropylthio-β-galactoside (IPTG), and then confirmed by SDS-PAGE. The results showed that the recombinant protein generated a band with a relative molecular mass of approximately 40 kDa (marked by red arrows; **Figure 4**) with IPTG treatment. The measured relative molecular mass was in accordance with the theoretical value. However, the control group without IPTG did not express this protein. The recombinant protein was purified and the enzyme activity was determined. First, the linear regression equations of quercetin (*y* = 0.002*x*, *R* ^2^ = 0.993) and kaempferol (*y* = 0.0175*x*, *R*2 = 0.998) were established. Then, the measured absorbance values were substituted into the linear regression equation. Finally, the reaction system produced 390 μg (1.29 μmol) of quercetin and 145.7 μg (0.51 μmol) of kaempferol within 20 min. In conclusion, the FLS activities were 1.29 ×10^–3^ IU/μL and 0.51×10^–3^ IU/μL using dihydroquercetin and dihydrokaempferol as substrate, respectively.

### *In vivo* localization of IbFLS1

**Figure 5:**
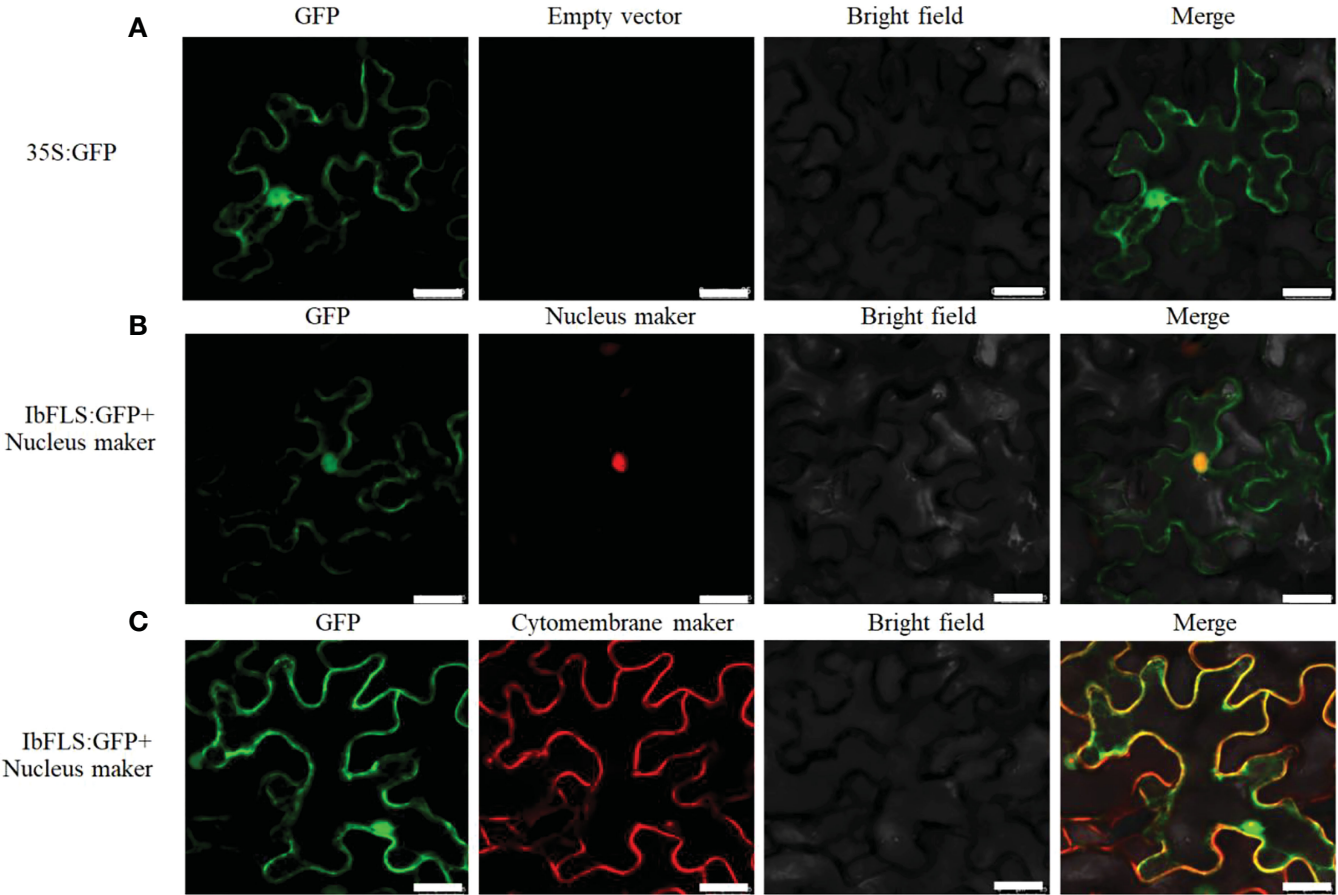
Subcellular localization of IbFLS1 proteins. Green fluorescence, GFP; red fluorescence, nucleus and cytomembrane makers; yellow fluorescence, combined signals. Scale bars: 25 μm.

The recombinant plasmid IbFLS1:GFP, along with the nucleus (or cytomembrane) localization marker, was infiltrated into tobacco leaves. Compared with empty vector (**Figure 5A**), under different excitation light irradiation, the IbFLS1:GFP fusion protein emitted green fluorescence, while the nucleus localization marker emitted red fluorescence (**Figure 5B**). After combining the two images, we found that the red fluorescence and the green fluorescence merged together and created yellow signals, indicating that the IbFLS1:GFP fusion protein was localized in the nucleus. As shown in **Figure 5C**, we found that the fluorescence from the IbFLS1:GFP fusion protein and from the cytomembrane localization maker overlapped, suggesting that the IbFLS1:GFP fusion protein was also localized in the cytomembrane. In summary, IbFLS is both a nuclear and a cytomembrane protein.

### Identification of transgenic sweet potato plants

**Figure 6:**
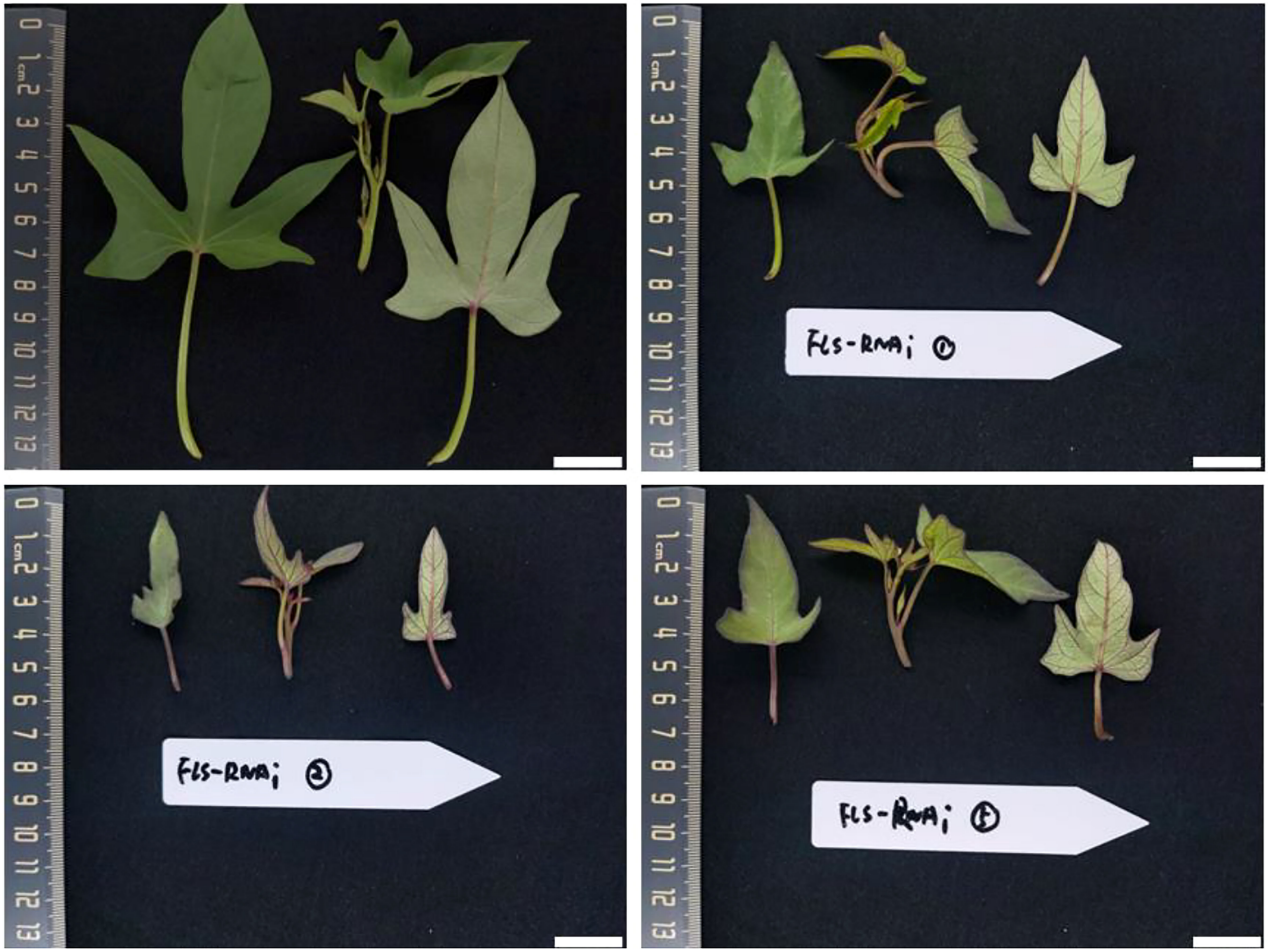
Leaves of the transgenic sweet potato plant. Leaves were collected 120 days after transplanting. Scale bars: 2 cm.

**Figure 7:**
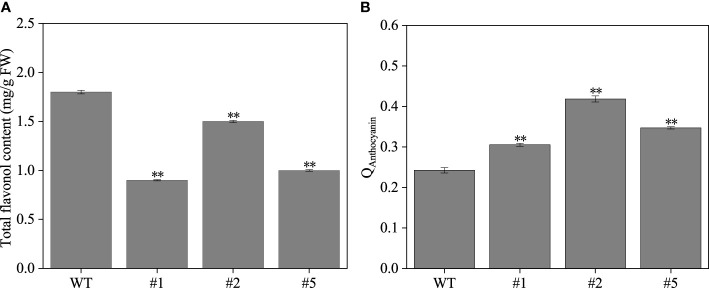
The flavonol **(A)** and anthocyanin **(B)** content in transgenic sweet potato leaves. Leaves were collected 120 days after transplanting. The significance analysis was done with wild type as the control. We used a one-way analysis of variance. **Significant differences within the different groups (*p* < 0.01).

**Figure 8:**
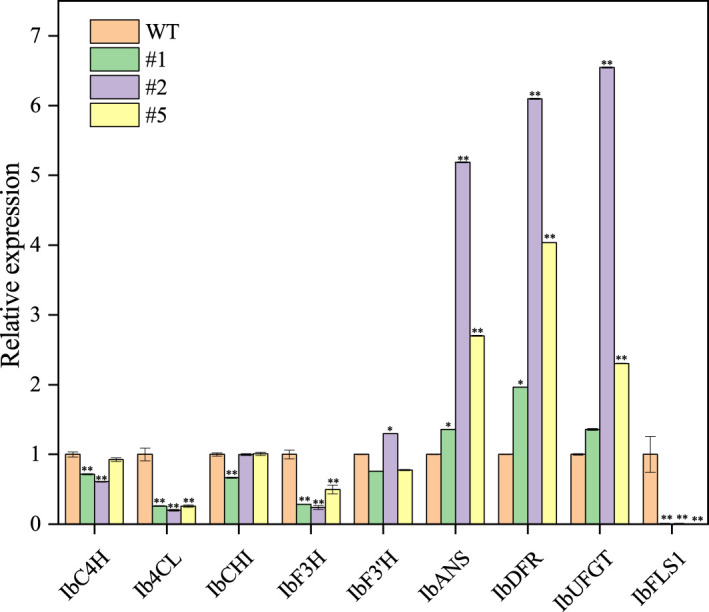
Expression levels of anthocyanin synthesis-related genes in transgenic sweet potato leaves. Leaves were collected 120 days after transplanting. The significance analysis was done with WT as the control. We used a one-way analysis of variance. ** Significant differences within the different groups (*p* < 0.01).

**Figure 9:**
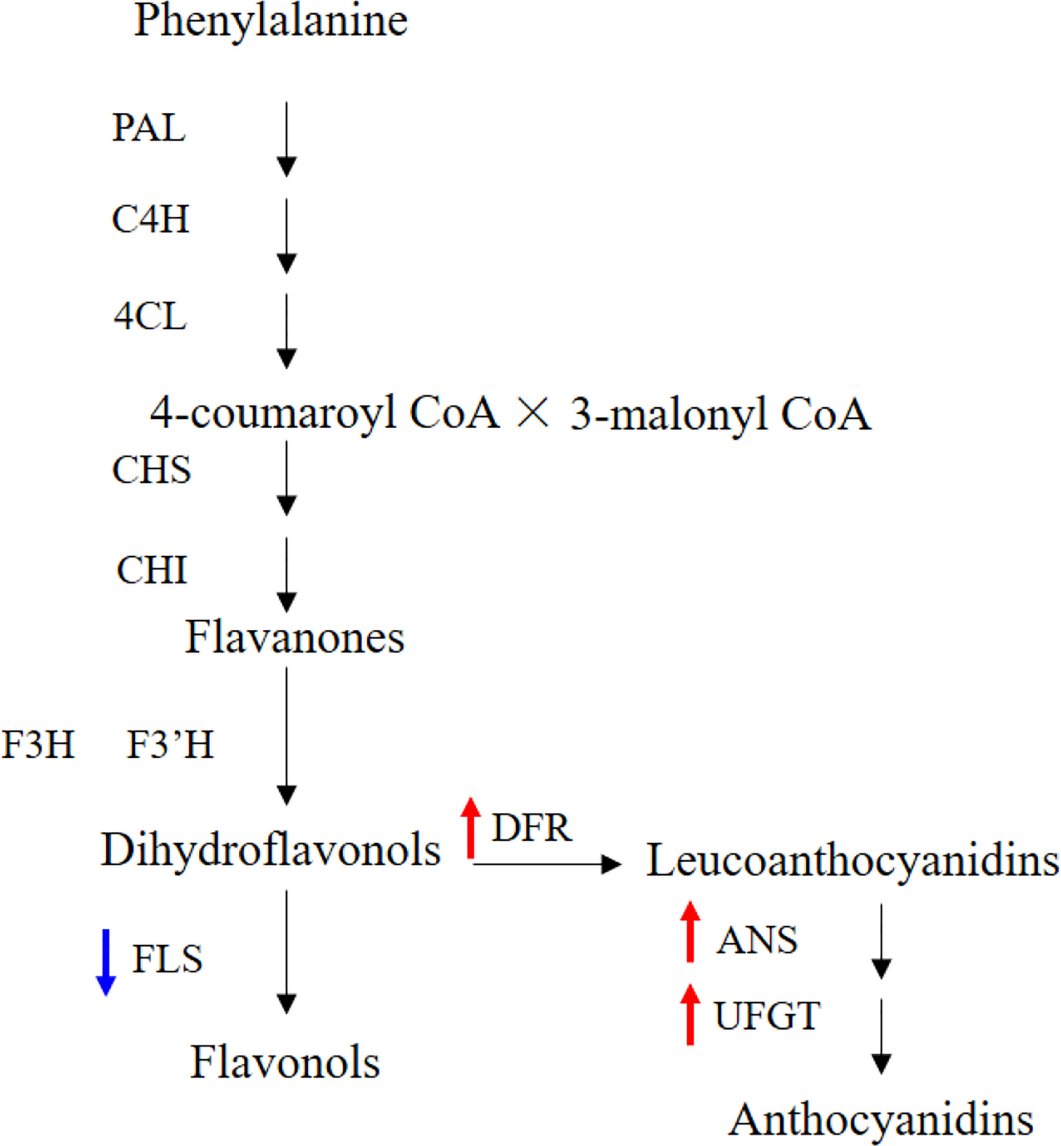
Silencing of IbFLS1 regulates anthocyanin metabolism pathway in transgenic sweet potato leaves. Blue arrows represent downregulated gene expression, while red arrows represent upregulated gene expression.

The IbFLS1:RNAi vector was transferred into the calli of ‘Xuzi 8’ *via A. tumefaciens* EHA105. After screening the hygromycin selection medium, more than 10 transgenic sweet potato lines were obtained. Compared with wild-type plants, the leaves of transgenic lines had significant anthocyanin pigmentation (**Figure 6**). Silencing of *IbFLS1* expression by RNA interference suppressed flavonol accumulation but promoted anthocyanin accumulation in transgenic sweet potato leaves (**Figure 7**). Furthermore, qRT-PCR analysis showed that the expression level of *IbFLS1* was significantly reduced. Similarly, the expression levels of other key genes (*C4H*, *4CL*, *CHI*, *F3H*, and *F3′H*) involved in the upstream pathway of anthocyanin biosynthesis were also decreased, but those of structural genes (*DFR*, *ANS*, and *UFGT*) related to the downstream pathway of anthocyanin biosynthesis were increased (**Figures 8**, **9**; primers are listed in **Supplementary Table S1**).

## Discussion

Transcriptome sequencing is a fast and efficient method of obtaining a large number of gene fragments. It plays an important role in gene discovery, transcription analysis, and molecular marker development without reference genomes (Grabherr et al., 2011; Wang et al., 2011). It also helps researchers uncover key candidate genes in biological metabolic pathways (Li et al., 2021). In this study, using sweet potato transcriptome data, a key candidate gene (*IbFLS1*) that may be involved in flavonol formation was screened out. Afterwards, the *IbFLS1* gene was cloned from sweet potato for the first time (to the best of our knowledge). Sequence comparison showed that the IbFLS1 protein is highly similar to FLSs from other known plants, with the typical HxDxnH motifs binding to ferrous ions and the RxS motifs binding to 2-oxoglutarate (**Figure 1B**). The phylogenetic tree grouped IbFLS1 with dicotyledonous plants, such as *Petunia hybrida*, *Nicotiana tabacum*, and *Solanum tuberosum*, but it was far from monocotyledonous plants, such as *Brachypodium distachyon* and *Sorghum bicolor* (**Figure 2**). These results indicated that FLS proteins in monocotyledonous plants and dicotyledonous plants are dissimilar and that IbFLS1 is more closely related to FLSs in dicotyledonous plants. Therefore, it was speculated that it is likely that *FLS* genes appeared after the evolution of monocotyledonous and dicotyledonous plants.

qRT-PCR analysis demonstrated that the expression of *IbFLS1* was tissue-specific and was highest in YL, while lower in stems, PRs, and SRs. This result is consistent with previous reports that in *Vitis vinifera* activity of *FLS* is highest in young tissues (Fujita et al., 2006), and that this helps to protect young tissues from UV-B damage (Zhang et al., 2015). Since the expression level of *IbFLS1* was lower in the SRs of PFSP, it was supposed that the SR is not the main organ of flavonol accumulation. Due to the anthocyanin-rich SRs of PFSP, the synthesis of flavonols is relatively lower. This phenomenon is in line with previous reports that *FLS* and *DFR* compete for common substrates, and that a low expression of *FLS* makes flavonoid synthesis flow to an anthocyanin biosynthetic pathway (Luo et al., 2016).

Wellmann et al. (2002) reported that *FLS* in *Citrus unshiu* had a different *K_m_* for converting dihydrokaempferol (*K_m_* 45 μmol/L) and dihydroquercetin (*K*_m_ 272 μmol/L) to the corresponding flavonols. In *Zea mays*, dihydrokaempferol (*K*_m_ 58 μmol/L) is the preferred substrate, rather than dihydroquercetin (*K*_m_ 151 μmol/L) for ZmFLS1 (Ferreyra et al., 2010). The catalytic activity of FtFLS1 was detected by thin-layer chromatography (TLC) and spectrophotometric assays, and dihydroquercetin, which had a higher specific activity than dihydrokaempferol, was the predominant substrate of FtFLS1 (Li et al., 2012). This result might raise the possibility that rutin in *Fagopyrum tataricums* may be synthesized from the hydroxylation of dihydrokaempferol to dihydroquercetin by *F3′H*, and oxidation of dihydroquercetin to quercetin, then glycoylation of quercetin to rutin (Li et al., 2015). In our study, using a similar method to that previously mentioned, the recombinant IbFLS1 was more effective at converting dihydroquercetin to quercetin than dihydrokaempferol to kaempferol, and had a specific activity similar to that of FtFLS1 (Li et al., 2015). Taken together, these results verified that IbFLS1 belongs to a flavonol synthase.

Sweet potato is a plant of the family Convolvulaceae originating from Central and South America. It has been widely cultivated as a food crop around the world due to its various advantages. The ornamental characteristics of the sweet potato have long been overlooked; normally, people only focus on the underground edible parts rather than the colorful flowers, leaves, and branches. Nowadays, the sweet potato has become an ornamental plant of great value and established a new use for germplasm resources. In this study, we have developed special transgenic sweet potato plants by RNA interference on *IbFLS1* expression. Silencing of *IbFLS1* increased the anthocyanin content and the reduced flavonol content of transgenic plant leaves (**Figure 6**). In conclusion, our work highlights potential methods for regulating anthocyanin and flavonol concentration of sweet potato by metabolic engineering.

## Data availability statement

The original contributions presented in the study are included in the article/**Supplementary Materials**, further inquiries can be directed to the corresponding author/s.

## Author contributions

MK and QL conceived and designed the study. QL conducted the experiments. MK performed the experiments and wrote the manuscript. MA and CL revised the manuscript. CL, WS, YS, and WT provided help with the experiments. CL, YZ, XW, HY, and RG collected and analyzed the data. All authors contributed to the article and approved the submitted version.
