# Supplementary material for: Identification and functional characterization of a flavonol synthase gene from sweet potato [Ipomoea batatas (L.) Lam.]
Source: Front Plant Sci. 2023 May 10;14:1181173. doi: 10.3389/fpls.2023.1181173 (PMC10206235; doi:10.3389/fpls.2023.1181173)
Supplement: Supplementary file 1 [file Table_1.docx]

**Supplementary Table S1** The primers used in the study

| *Primer* | *Sequence* | *Description* |
| --- | --- | --- |
| IbFLS-1-F | 5’-ATGGAGGTGGAGAGAGTGCA-3’ | Gene specific primer |
| IbFLS-1-R | 5’-TTACTGAGGAAGCTTGTTCAGCT-3’ |  |
| IbFLS-2-F | 5’-GCCTAATGAGGTGATCCGGG-3’ | Primer for qRT-PCR |
| IbFLS-2-R | 5’-CAACCCCTCTTCCCATCCAC-3’ |  |
| IbFLS-3-F | 5’-CATG catatg GAGGTGGAGAGAGTGCAA-3’  (small letters represent *Nde*I restriction site) | Primer for pET-28a(+) construction |
| IbFLS-3-R | 5’-CATGggatccTCACTTCTGAGGAAGCTTGTTCA-3’  (small letters represent *Bam*HI restriction site) |  |
| IbFLS-4-F | 5’-GAgtcgacATGGAGGTGGAGAGAGTGC-3’  (small letters represent *Sal*I restriction site) | Primer for pCAMBIA1301S-GFP construction |
| IbFLS-4-R | 5’-GGactagtACCTGAGGAAGCTTGTTCAGC-3’  (small letters represent *Spe*I restriction site) |  |
| FLS1i-F(*Kpn*I) | 5’-GAggtaccGGAGGTGGAGAGAGTGCAAG-3’  (small letters represent *Kpn*I restriction site) | Primer for FS area of IbFLS-RNAi clone |
| FLS1i-R(*Cla*I) | 5’-TCatcgatCCCCACTCTCTGCTCGCCTC-3’  (small letters represent *Cla*I restriction site) |  |
| FLS1i-F(*BamH*I) | 5’-CGggatccGGAGGTGGAGAGAGTGCAAG-3’  (small letters represent *BamH*I restriction site) | Primer for RS area of IbFLS-RNAi clone |
| FLS1i-R(*Xho*I) | 5’-GGActcgagCCCCACTCTCTGCTCGCCTC-3’  (small letters represent *Xho*I restriction site) |  |
| IbC4H | 5’-TTGTCGCCATTGTTGTGTCCAAG-3’ | Primer for qRT-PCR |
|  | 5’-CTTGGCGTAATCGGTGAGATTCC-3’ |  |
| Ib4CL | 5’-CAAAGAACTCATCAAATTCAAAGG-3’ | Primer for qRT-PCR |
|  | 5’-AACCACAAACGCAACAGG-3’ |  |
| IbCHI | 5’-AGTATTCGGAGAAGGTGTCG-3’ | Primer for qRT-PCR |
|  | 5’-GGCATTGAACCCTCTTTGG-3’ |  |
| IbF3H | 5’-TCATCGTTTCCAGCCATCTC-3’ | Primer for qRT-PCR |
|  | 5’-ACTTTTCCGTTACTGCCCTCC-3’ |  |
| IbF3'H | 5’-AGAAACCGCACCAGTCGATT-3’ | Primer for qRT-PCR |
|  | 5’-CAATATGTTTGGCGCCGGAG-3’ |  |
| IbDFR | 5’-GCTCTTCTCATCATACAACCATCC-3’ | Primer for qRT-PCR |
|  | 5’-AGGCAAGTCCTTTTCAATACCC-3’ |  |
| IbANS | 5’-CTCTCCATTTGGCCTAAAACTCC-3’ | Primer for qRT-PCR |
|  | 5’-TCTCCAGCCTTCCTTCCTCTAAC-3’ |  |
| IbUFGT | 5’-TTCGTAGTTTAGTCGCCGCC-3’ | Primer for qRT-PCR |
|  | 5’-CCGATCGATACTTCTGACATTCC-3’ |  |
